# Mxene/alginate composites for lead and copper ion removal from aqueous solutions

**DOI:** 10.1039/c9ra05251h

**Published:** 2019-09-16

**Authors:** Yanjie Dong, Dashen Sang, Chengdong He, Xinfeng Sheng, Longwen Lei

**Affiliations:** Anhui Provincial Laboratory of Optoelectronic and Magnetism Functional Materials, College of Chemistry and Chemical Engineering, Anqing Normal University Anqing 246011 People's Republic of China dongyjaqtc@126.com

## Abstract

Mxene has been widely used as a sorbent to remove heavy metal ions from sewage due to its unique two-dimensional layered structure and abundant oxygen-containing groups. However, Mxene has a relatively limited adsorption capacity for metal ions possibly due to the limited adsorption active sites. Herein, we reported novel Mxene/alginate composites for lead and copper ion removal from wastewater. The Mxene/alginate composites prepared in this study not only enhance the chelation ability of the lead and copper ions, but also accelerate the ion transport efficiency. The combined advantages of high adsorption capacity and short equilibrium time enable the Mxene/alginate composites to achieve the maximum adsorption capacity for Pb^2+^ and Cu^2+^ at 382.7 and 87.6 mg g^−1^, respectively, and reach the adsorption equilibrium in 15 min. We believe that the composites developed in this study can open a new avenue for designing high adsorption capacity and high efficiency adsorbents.

## Abstract 2

Studies on Mxene/alginate composite adsorption have opened up a new avenue for designing adsorbents possessing high adsorption capacity and high efficiency.

## Graphical abstract

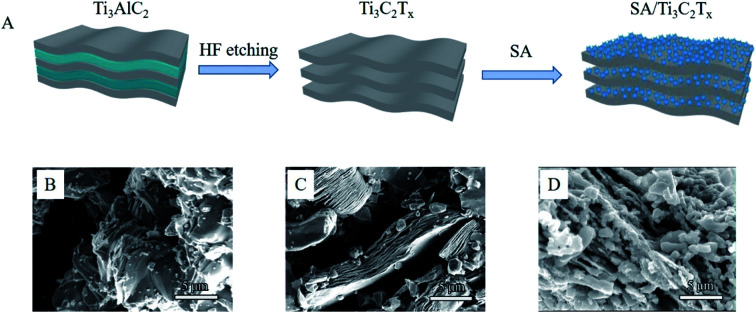

## 1. Introduction

With the rapid development of industrial economy, heavy metal pollution has become one of the most serious environmental problems in recent decades.^1^ The treatment of heavy metals is of special concern given their toxicity and persistence in the environment. There is no doubt that it poses a great threat to the ecological environment and human health.^1,2^ Thus, the ability to efficiently remove heavy metal ions from water has gradually become the focus of environmental researchers.

In recent years, with the development of wastewater technologies, such as adsorption, membrane filtration, chemical precipitation and ion exchange, great progress has been made in the treatment and purification of heavy metals in wastewater.^3–5^ Among these methods, adsorption is regarded as a promising method given its simple operation, high treatment efficiency, low-cost, and excellent recyclability.^6^

Mxene, a new graphene-like 2D material, has a wide range of applications in energy storage, catalysis and sewage treatment due to its unique layered structure and hydrophilic surface. Shahzad *et al.* synthesized Ti_3_C_2_T_*x*_ Mxene nanosheets for an efficient copper removal from water.^7^ Ying *et al.* fabricated 2D Ti_3_C_2_T_*x*_ with regular structure by HF etching, which showed superior performance for the adsorption of chromium.^8^ These Mxene materials have shown potential in the treatment of heavy metals. However, Ti_3_C_2_T_*x*_ as a single adsorption material has a relatively limited adsorption capacity for heavy metal ions due to the limited active sites.

In this study, we designed and fabricated Mxene/alginate composites to overcome the problem of limited adsorption capacity in a single Mxene. A large number of amino and carboxyl groups in alginate have good chelating ability for heavy metal ions, which can significantly increase the adsorption capacity of the composites. In addition, the 2D lamellar structure of the composites can greatly improve the transport efficiency of heavy metal ions in the adsorption process and significantly shorten the time required to reach the adsorption equilibrium. Here, we demonstrated that Mxene/alginate composites are indeed a promising sorbent for heavy metal treatment.

## 2. Experimental

### 2.1. Reagents

Sodium alginate, nitric acid, calcium nitrate, hydrofluoric acid, titanium aluminum carbide (Ti_3_AlC_2_), lead nitrate (Pb(NO_3_)_2_), and copper nitrate trihydrate (Cu(NO_3_)_2_·3H_2_O) were purchased from the Shanghai Aladdin Biochemical Technology Co., Ltd. All reagents were of analytical grade and were used without further purification, and deionized water was used as a solvent throughout this study.

### 2.2. Apparatus

Scanning electron microscopy (SEM, FEI Sirion 200, Eindhoven, the Netherlands) was used to characterize the surface morphology of samples. Fourier transform infrared spectroscopy (FT-IR, Thermo Nicolet 6700, Waltham, USA) and X-ray photoelectron spectroscopy (XPS, Shimadzu Axis-Ultra DLD, Tokyo, Japan) were performed in this study to acquire the material composition analysis. The metal ion concentrations before and after adsorption were confirmed by inductively coupled plasma-optical emission spectrometry (ICP-OES, PerkinElmer Optima 8000, Waltham, USA).

### 2.3. Fabrication of Ti_3_C_2_T_*x*_

For the fabrication of Ti_3_C_2_T_*x*_, 2 g Ti_3_AlC_2_ was first added into 30 mL HF (40% aqueous solution) and stirred for 12 h. Then, the mixture was washed with deionized water several times until a pH value of 5 was achieved. Finally, Ti_3_C_2_T_*x*_ was obtained by drying in a vacuum oven at 80 °C for 24 h.

### 2.4. Preparation of Mxene/alginate composites

0.16 g Ti_3_C_2_T_*x*_ and varying amounts of sodium alginate (68, 106, 160, 240, 370, and 640 mg) were first added into 20 mL deionized water, and the mixture was stirred with a magnetic stirrer for 6 h. Then, the mixture was placed into an ultra-low temperature refrigerator for 12 h. Finally, the Mxene/alginate composites were obtained by freeze-drying under vacuum for 24 h. The cross-linked Mxene/alginate composites were also prepared in this study under the same condition using calcium nitrate (0.2 M) as a cross-linker.

### 2.5. Adsorption experiment

To complete the adsorption test, Mxene/alginate composites were first soaked in 50 mL of 1.5 mM Pb^2+^ and Cu^2+^ solutions, respectively. The mixture was filtered after stirring for 15 min. The unextracted Pb^2+^ and Cu^2+^ in the solution were confirmed by ICP-OES. Each adsorption and desorption for Pb^2+^ and Cu^2+^ was performed three times in parallel, and the corresponding ion concentration depended on the average of the three parallel experiments. The corresponding adsorption capacity (*Q*, mg g^−1^) and the percentage of adsorbed metal ions (*η*, %) were calculated using the following formulae:
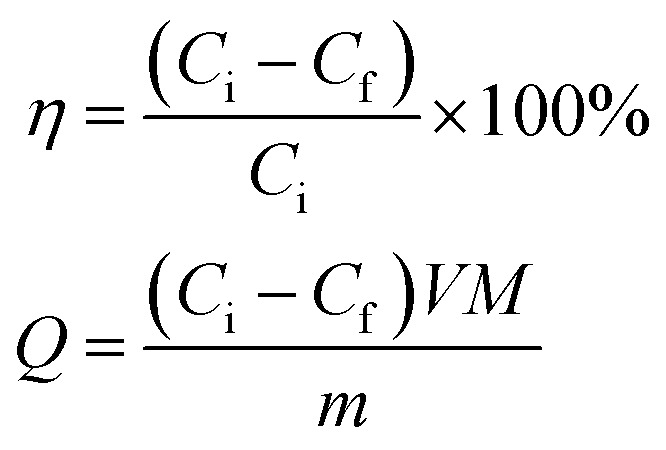
where *C*_i_ and *C*_f_ represent the concentration of metal ions in solution before and after adsorption, respectively (mM); *V* is the volume of the solution (L); *M* is the molar mass of the metal ions (g mol^−1^); and *m* is the mass of the Mxene/alginate composite (g).

## 3. Results and discussion

### 3.1. Material characterization

**Fig. 1:**
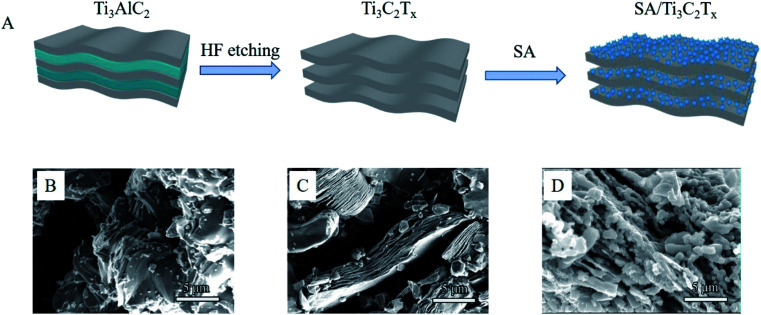
(A) Schematic of the preparation of the Mxene/alginate composites; (B) surface morphology of Ti_3_AlC_2_; (C) surface morphology of Ti_3_C_2_T_*x*_; (D) surface morphology of the Mxene/alginate composites.

The preparation process of the Mxene/alginate composites is shown in Fig. 1A. The original Ti_3_AlC_2_ (MAX) shows an irregular morphology (Fig. 1B). After etching with hydrofluoric acid, Ti_3_C_2_T_*x*_ (Mxene) presents a typical two-dimensional organ-like morphology (Fig. 1C). When alginate is added to the Ti_3_C_2_T_*x*_, alginate tends to occupy the interlayer, and then cover a part of the surface of Ti_3_C_2_T_*x*_. Compared with pure Ti_3_C_2_T_*x*_, the Mxene/alginate composites have a rougher surface (Fig. 1D), which provides a superior environment for Pb^2+^ and Cu^2+^ adsorption.

**Fig. 2:**
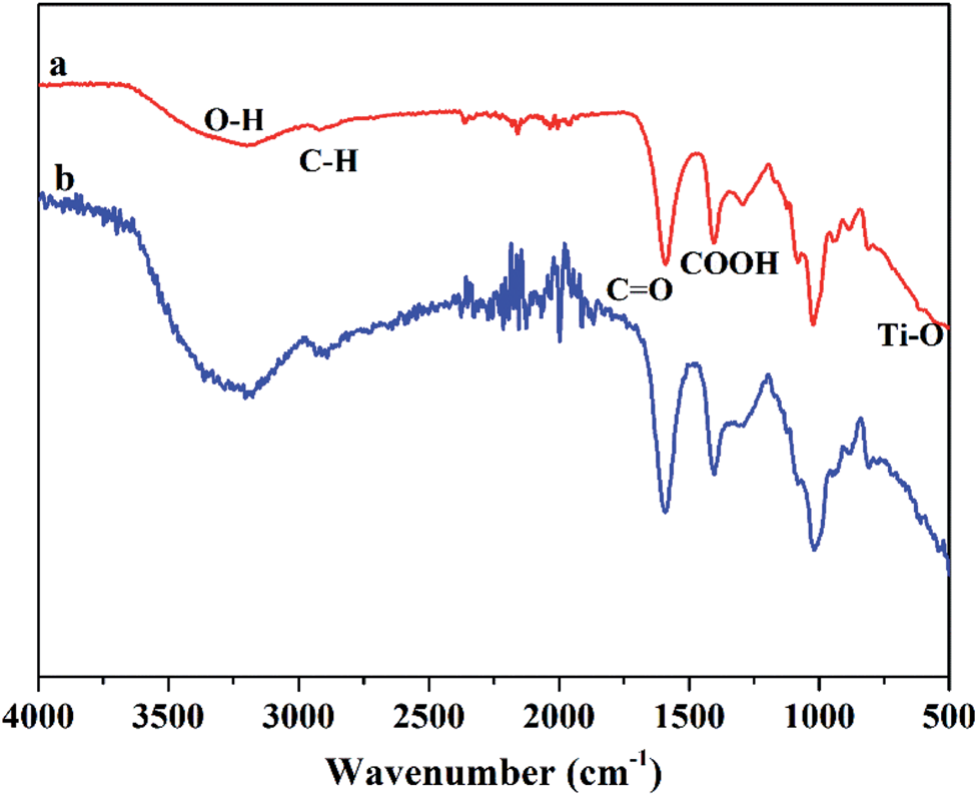
FT-IR spectra of (a) Mxene/alginate composites and (b) sodium alginate.

In addition, FT-IR analysis is conducted to explore the functional groups of sodium alginate and the Mxene/alginate composites. As shown in Fig. 2, the broad adsorption peak at 3210 cm^−1^ belongs to the O–H stretching vibration. The adsorption peak at 2908 cm^−1^ and 1026 cm^−1^ are attributed to the aliphatic C–H stretching vibration and C–O stretching vibration in sodium alginate. The peaks at 1590 and 1399 cm^−1^ can be assigned to the vibrational modes of C=O and C–O in the carboxyl group.^9,10^ Compared with the infrared spectrum of sodium alginate, there is a new adsorption peak corresponding to the Ti–O stretching vibration at 596 cm^−1^ in the spectra of the Mxene/alginate composites, which confirms that sodium alginate was successfully incorporated into the Mxene.^11^ Moreover, the peak shift from 3210 cm^−1^ in the sodium alginate to 3196 cm^−1^ in the Mxene/alginate composites suggest the formation of hydrogen bonds between sodium alginate and Mxene.^12^

### 3.2. Effect of alginate ratio in Mxene/alginate composites

**Fig. 3:**
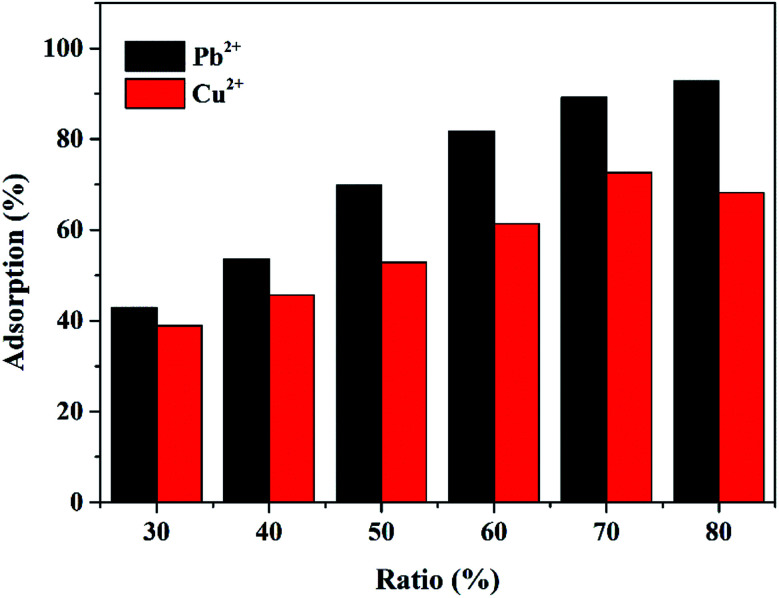
Effect of alginate ratio in Mxene/alginate composite on Pb^2+^ and Cu^2+^ adsorption.

In order to evaluate the influence of the ratio of sodium alginate on the ability of the Mxene/alginate composites to absorb Pb^2+^ and Cu^2+^, a series of Mxene/alginate composites with different ratios of sodium alginate were prepared in this study. As shown in Fig. 3, the adsorption efficiency of the Mxene/alginate composite for Pb^2+^ and Cu^2+^ increased with an increase in the ratio of sodium alginate and then remained relatively stable. This result can be explained by the large number of amino and carboxyl groups in sodium alginate, which can effectively chelate Pb^2+^ and Cu^2+^, and significantly improves the adsorption efficiency of the Mxene/alginate composites. However, when the ratio of sodium alginate continues to increase, sodium alginate not only fills the Ti_3_C_2_T_*x*_ layer, but also covers the Ti_3_C_2_T_*x*_ surface, thus possibly making Ti_3_C_2_T_*x*_ lose part of its adsorption capacity. Therefore, the ratio of sodium alginate in 30–70% range, as the ratio of sodium alginate increase, the adsorption efficiency of the Mxene/alginate composites to Pb^2+^ and Cu^2+^ increase, the ratio of sodium alginate over 70%, the adsorption efficiency of the Mxene/alginate composites to Pb^2+^ and Cu^2+^ decrease.

### 3.3. Effect of Mxene/alginate composite sorbent dosage

**Fig. 4:**
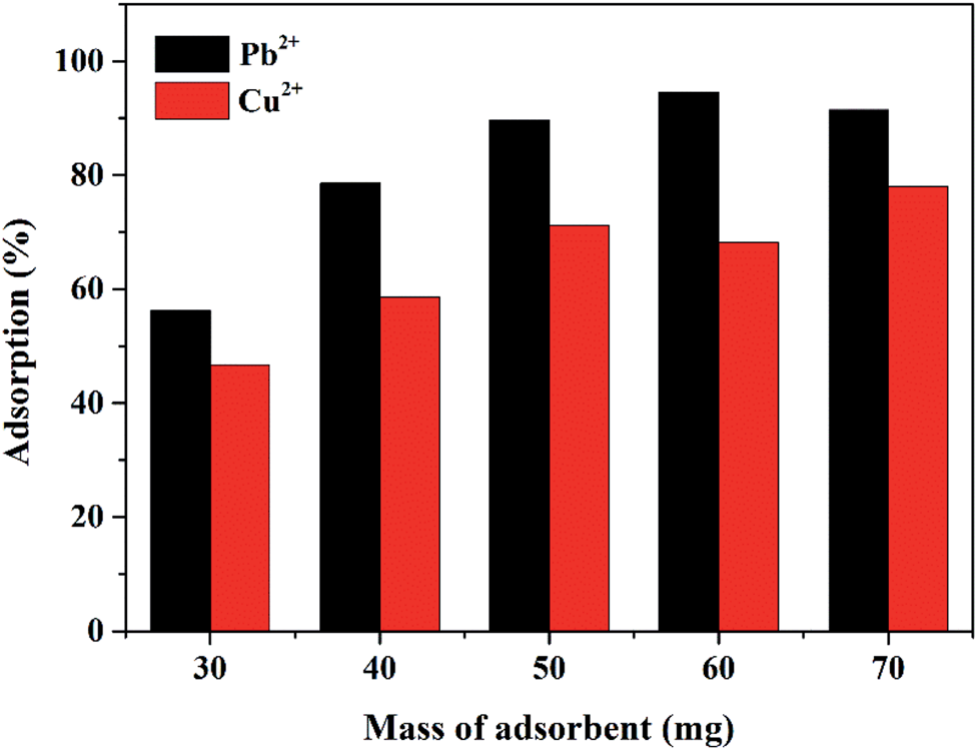
Effect of composite sorbent dosage on Pb^2+^ and Cu^2+^ adsorption.

To obtain the superior adsorption performance, the Mxene/alginate composite sorbent dosage was optimized in this study before exploring the adsorption capacity for Pb^2+^ and Cu^2+^. The corresponding results are presented in Fig. 4. When the composite adsorbent dosage was small (<50 mg), the adsorption efficiency increased significantly with the increase in the adsorbent dosage. However, when the adsorbent dosage was equal to or more than 50 mg, there was a modest increment in the adsorption efficiency. This could be possibly due to the adequate adsorption active sites provided by the Mxene/alginate composite. Thus, 50 mg of the Mxene/alginate composite adsorbent was used throughout the experiment.

### 3.4. Effect of pH

**Fig. 5:**
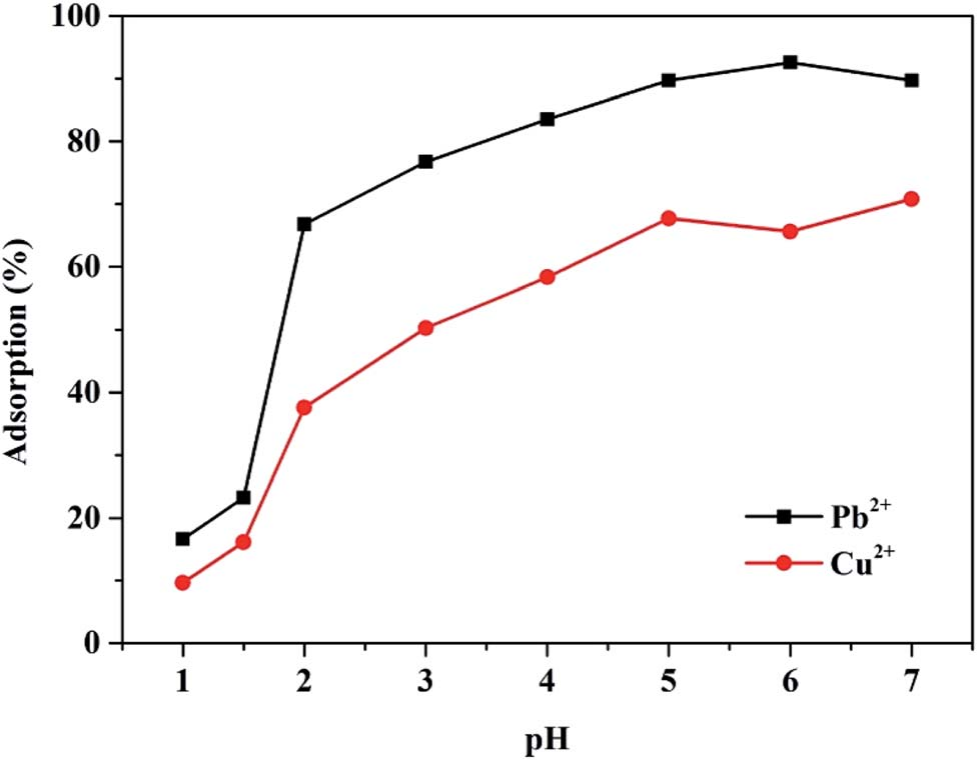
Effect of pH on the adsorption of Pb^2+^ and Cu^2+^.

The pH value of the solution is an important factor that influences the adsorption of heavy metals. Considering that the industrial heavy metal ion wastewater is usually acidic, the pH range was set to pH values of 1 to 7 in this study. As shown in Fig. 5, the adsorption efficiency of the Mxene/alginate composite to Pb^2+^ and Cu^2+^ was extremely limited when the aqueous solution was more acidic (pH < 1). As the acidity of the solution decreased, the adsorption efficiency of the Mxene/alginate composite to Pb^2+^ and Cu^2+^ was significantly improved. When the pH value reached a certain level (pH ≥ 5), the adsorption curve of the Mxene/alginate composite on Pb^2+^ and Cu^2+^ appeared to plateau, which indicated that the adsorption of the Mxene/alginate composite on Pb^2+^ and Cu^2+^ was stabilized. This experimental phenomenon can be explained by competitive adsorption. When the pH value is small, a lot of H^+^ and H_3_O^+^ ions in the solution occupied the active sites on the surface of the adsorbent. In addition to the increased pH value of the solution, the ability of the H^+^ and H_3_O^+^ ions to occupy the adsorption sites decreases, and the heavy metal ions with positive charges begin to occupy the adsorption sites instead. Thus, the adsorption capacity of the adsorbent to Pb^2+^ and Cu^2+^ increases.^10^

### 3.5. Effect of time and temperature

**Fig. 6:**
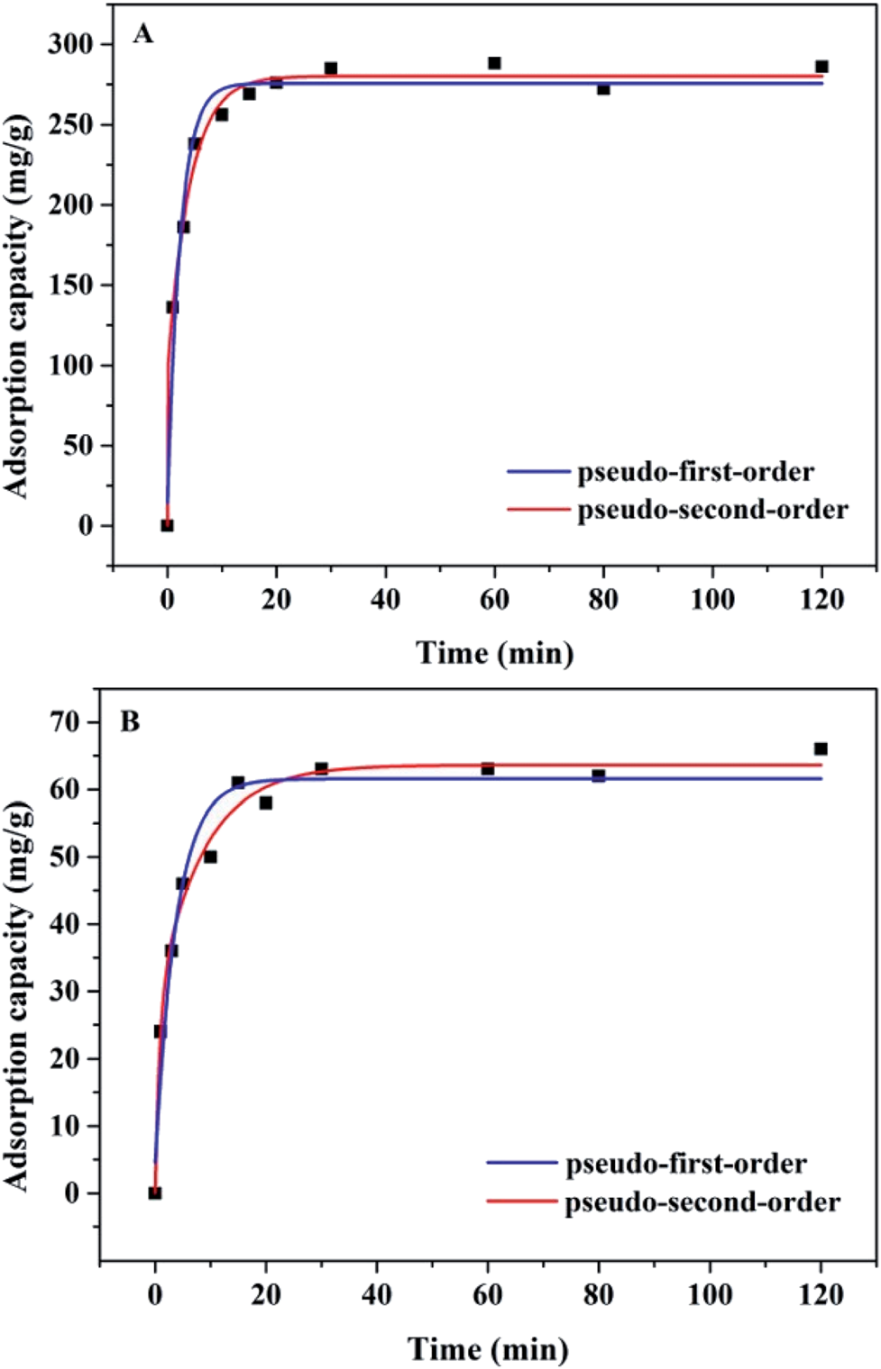
Adsorption kinetics studies of (A) Pb^2+^ and (B) Cu^2+^ adsorption.

The effect of time on the adsorption of Pb^2+^ and Cu^2+^ are shown in Fig. 6. It is obvious that the adsorption rate of Pb^2+^ and Cu^2+^ onto the Mxene/alginate composite was fast during the initial 15 min, which can adsorb 86.7% Pb^2+^ and 63.5% Cu^2+^, respectively. This is attributed to the abundant adsorption groups provided by sodium alginate and Ti_3_C_2_T_*x*_. Furthermore, the short equilibrium time (15 min in this study) was obtained due to the 2D layered structure provided by Ti_3_C_2_T_*x*_. This was conducive to the intercalation and diffusion of the ions, thus accelerating the transport efficiency of Pb^2+^ and Cu^2+^. As the reaction time progressed, the adsorption sites in the Mxene/alginate composites were gradually occupied and the adsorption curve appeared as a plateau, indicating that the adsorption process reached equilibrium. In addition, in order to further study the kinetic behavior of adsorption, pseudo-first-order and pseudo-second-order models were adopted to match the relevant experiment data. The corresponding results shown in Fig. 6 and Table 1 illustrate that there is a better match for the pseudo-second-order kinetic model based on the values of *R*^2^, which suggests that chemisorption plays an important role in the adsorption process.^4,13^

**Table 1:** Kinetic parameters for Pb^2+^ and Cu^2+^ adsorption on the Mxene/alginate composites

Kinetic model	Formula	Parameters	Pb^2+^	Cu^2+^
Pseudo-first-order	*q*_t_ = *q*_e_(1 − exp(−*k*_1_*t*))	*q*_e_ (mg g^−1^)	275.6403	61.5735
		*k*_1_ (L min^−1^)	0.2599	0.1206
		*R* ^2^	0.9661	0.9559
Pseudo-second-order	*q*_t_ = *q*_e_(1 − 1/(1 + *q*_e_*k*_2_*t*))	*q*_e_ (mg g^−1^)	280.1126	63.6194
		*k*_2_ (L min^−1^)	0.0069	0.0064
		*R* ^2^	0.9897	0.9839

**Fig. 7:**
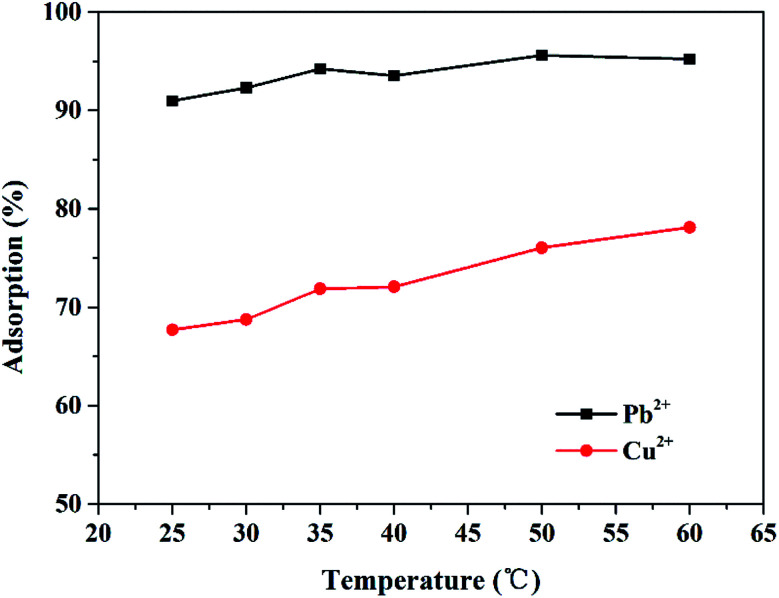
Effect of temperature on Pb^2+^ and Cu^2+^ adsorption.

In addition, the effect of temperature was also investigated in this study. As displayed in Fig. 7, the adsorption efficiency of Pb^2+^ and Cu^2+^ generally increased with the increase in temperature, which demonstrate that the adsorption of Pb^2+^ and Cu^2+^ by the Mxene/alginate composite is an endothermic process. However, it is not clear that the improvement in the adsorption efficiency for Pb^2+^ and Cu^2+^ is caused by temperature, indicating that the temperature had little influence on ion migration. It may be that the two-dimensional layered structure provided by Ti_3_C_2_T_*x*_ optimized the ion migration and intercalation, making the ion transport less dependent on temperature.

### 3.6. Maximum adsorption capacity

**Fig. 8:**
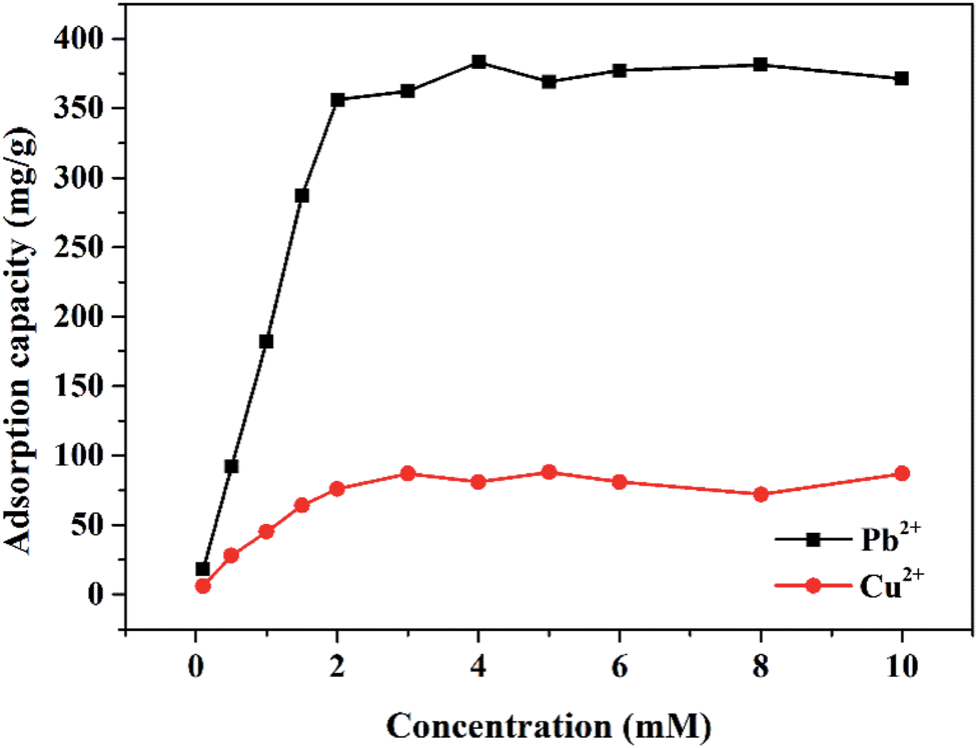
Effect of ion concentration on Pb^2+^ and Cu^2+^ adsorption.

The maximum adsorption capacity of the Mxene/alginate composite for Pb^2+^ and Cu^2+^ was investigated and is displayed in Fig. 8. Initially, the adsorption capacity was significantly improved with the increase in ion concentration. Subsequently, there was no significant change in the adsorption capacity with an additional increase in ion concentration possibly due to the lack of active sites for the excess ions. The maximum adsorption capacities of the Mxene/alginate composite for Pb^2+^ and Cu^2+^ in this study were 382.7 and 87.6 mg g^−1^, respectively, which were higher values than that of most reported adsorbents for the removal of Pb^2+^ and Cu^2+^ from water. The reported maximum adsorption capacities of adsorbents for the removal of Pb^2+^ and Cu^2+^ from water are summarized in Table 2.

**Table 2:** Maximum adsorption capacity of reported adsorbents for Pb^2+^ and Cu^2+^ removal from water

Adsorption material	Adsorbed ions	Maximum adsorption capacity (mg g^−1^)	Reference
Polydopamine microspheres	Pb^2+^	165.8	14
Amino functionalized magnetic graphene composite	Pb^2+^	28.0	15
Biochar-alginate capsule	Pb^2+^	263.2	16
Polyvinyl alcohol/polyacrylic acid double network gel	Pb^2+^	195.0	17
Polyaniline/calcium alginate composite	Pb^2+^, Cu^2+^	357.0 (Pb^2+^), 79.0 (Cu^2+^)	18
Silica modified calcium alginate–xanthan gum hybrid bead composite	Pb^2+^	18.9	19
Soy protein hollow microsphere material	Pb^2+^	235.6	20
Activated carbon–calcium alginate composite	Pb^2+^	15.7	21
Alginate–SBA-15 composite	Pb^2+^	222.2	22
Magnetic alginate beads	Pb^2+^	50.0	23
γ-Fe_2_O_3_ nanoparticles	Pb^2+^, Cu^2+^	69.0 (Pb^2+^), 34.0 (Cu^2+^)	24
Magnetic chitosan/graphene oxide imprinted Pb^2+^	Pb^2+^	79.0	25
Chitosan coated calcium alginate	Pb^2+^	106.9	26
Amino functionalized mesoporous silica	Pb^2+^	57.7	6
Hydroxyapatite/chitosan porous material	Pb^2+^	264.4	27
Calcite-poly(ethyleneimine) nanostructured rod	Pb^2+^	240	28
Nano-alumina	Pb^2+^	100.0	29
Calcium alginate/graphene oxide composite aerogel	Pb^2+^, Cu^2+^	368.2 (Pb^2+^), 98.1 (Cu^2+^)	4
Mxene/alginate composite	Pb^2+^, Cu^2+^	382.7 (Pb^2+^), 87.6 (Cu^2+^)	This work

**Fig. 9:**
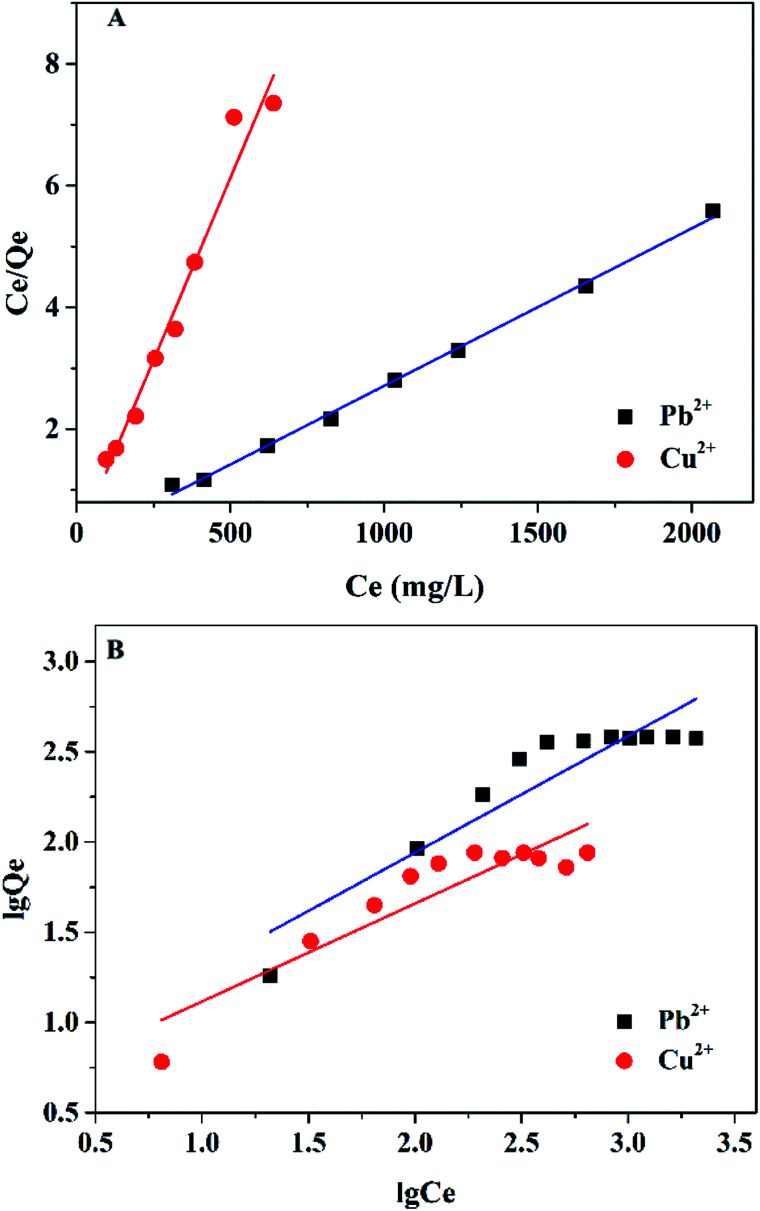
(A) Langmuir and (B) Freundlich adsorption isotherm studies on Pb^2+^ and Cu^2+^.

To acquire more accurate information about the interaction between the Mxene/alginate composite and heavy metal ions, Langmuir and Freundlich adsorption isotherm models were adopted in this study and are displayed in Fig. 9. The isotherm constants were calculated based on the experimental data and are presented in Table 3. The experimental results show that the Langmuir isotherm model exhibited a better correlation to describe the adsorption process compared with the Freundlich isotherm model, indicating that the adsorption process is more inclined to a monolayer adsorption.

**Table 3:** Isotherm parameters for Pb^2+^ and Cu^2+^ adsorption

Isotherm model	Formula	Parameters	Pb^2+^	Cu^2+^
Langmuir	*C*/*Q* = *C*/*Q*_e_ + 1/(*Q*_e_*b*)	*Q*_e_ (mg g^−1^)	380.56	83.52
		*b* (L mg^−1^)	0.067	0.011
		*R* ^2^	0.997	0.964
Freundlich	lg *Q* = lg *K* + 1/*n* lg *C*	*K* (L mg^−1^)	8.688	5.729
		*n*	1.528	2.728
		*R* ^2^	0.826	0.819

### 3.7. Adsorption mechanism

**Fig. 10:**
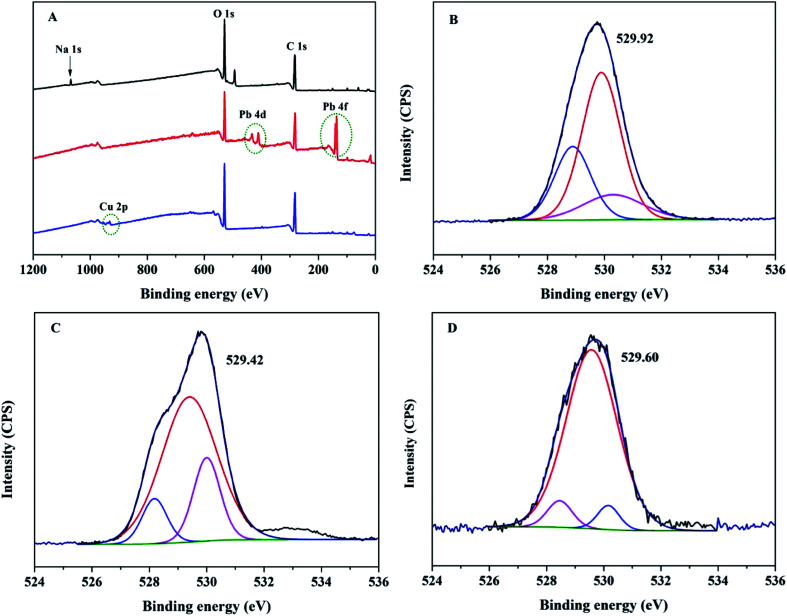
(A) X-ray photoelectron spectra of Mxene/alginate composites before and after adsorption; (B, C and D) O 1s spectra of Mxene/alginate composites (B) before, (C) after Pb^2+^ and (D) after Cu^2+^ adsorption.

**Fig. 11:**
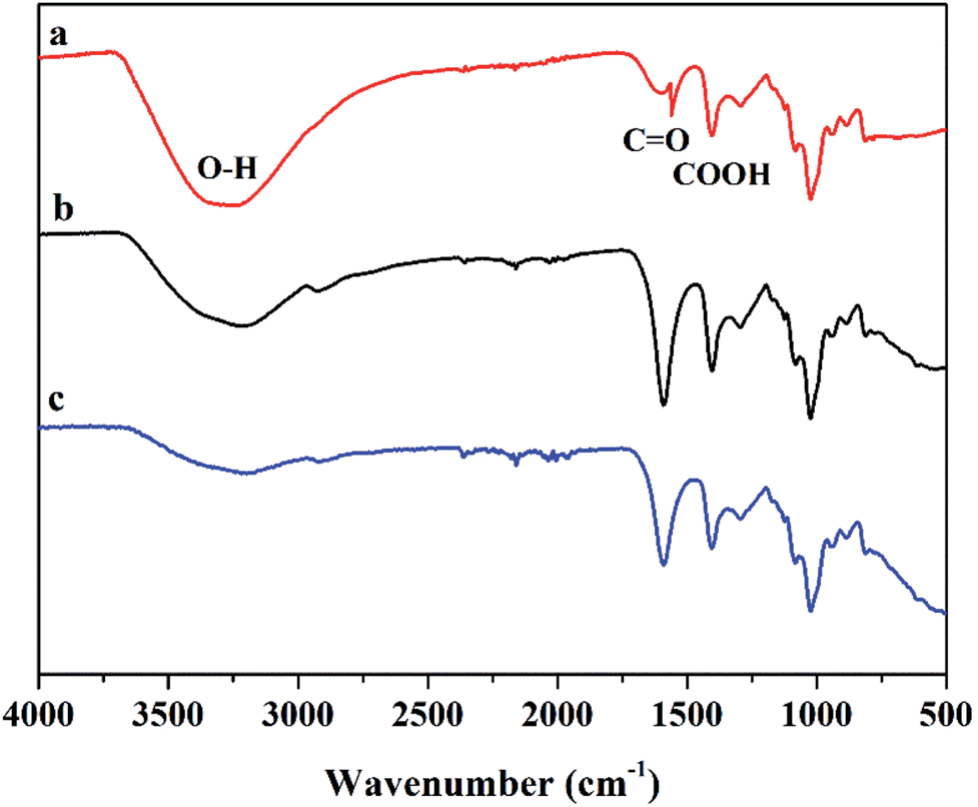
FT-IR spectra of (a) Mxene/alginate composite, (b) Mxene/alginate composite loaded Pb^2+^ and (c) Mxene/alginate composite loaded Cu^2+^.

To reveal the adsorption essence of the Mxene/alginate composites for Pb^2+^ and Cu^2+^, X-ray photoelectron spectra of Mxene/alginate composites before and after adsorption were investigated. The disappearance of the Na 1s peak with the appearance of the Pb 4d and Pb 4f (or Cu 2p) peaks after adsorption of Pb^2+^ (or Cu^2+^) suggests that ion exchange might be involved in the adsorption mechanism (Fig. 10A). To further explore the Mxene/alginate composite adsorption mechanism, the O 1s peak was analyzed before and after Pb^2+^ and Cu^2+^-loading (Fig. 10B–D). The experimental results showed that the binding energy of the oxygen-containing function groups decreased slightly from 529.92 to 529.42 and 529.60 eV after Pb^2+^ and Cu^2+^ adsorption, respectively. This result is consistent with the FT-IR results. The carboxylate peak of the composites shifted from 1410 to 1386 and 1398 cm^−1^ after the adsorption of Pb^2+^ and Cu^2+^ (Fig. 11), indicating that the chemical coordination of the oxygen atom of Mxene/alginate composites with Pb^2+^ and Cu^2+^ occurs during the adsorption process.

Based on the relevant XPS and FT-IR data analyses, a possible binding mechanism of the Mxene/alginate composites with Pb^2+^ and Cu^2+^ is a combination of the ion exchange and chemical coordination processes.

### 3.8. Regeneration study

**Fig. 12:**
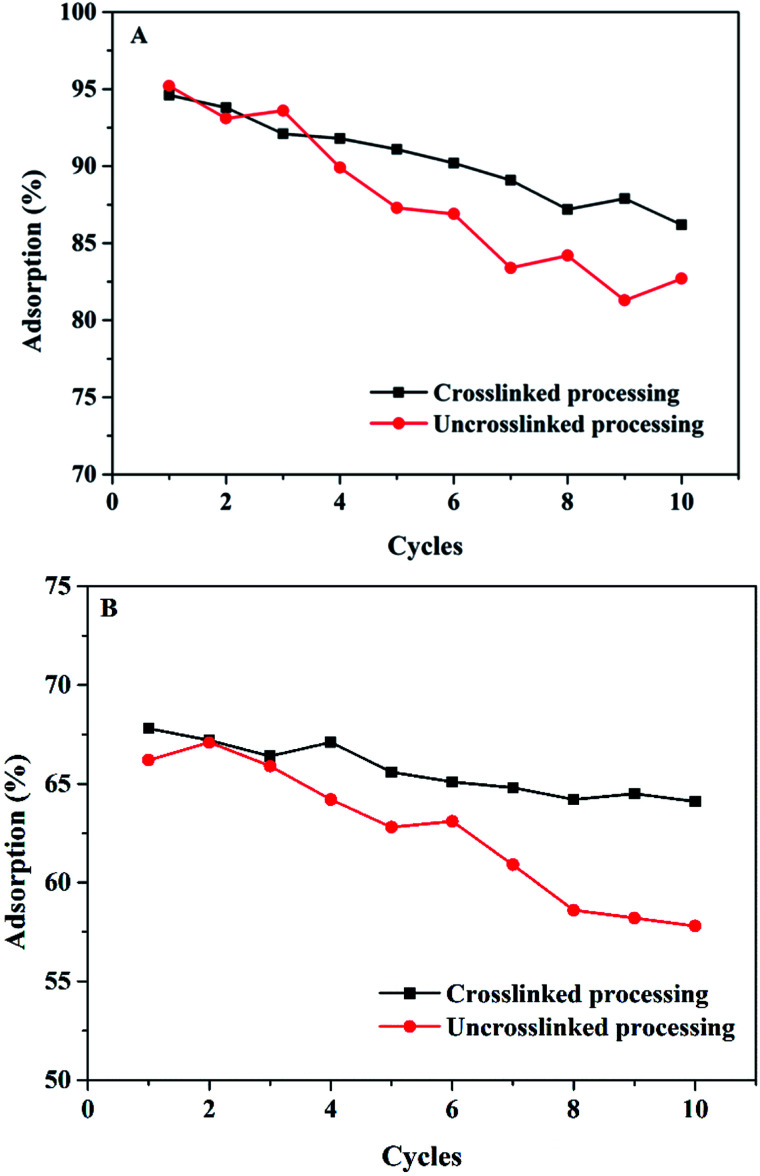
Regeneration study of the Mxene/alginate composites for (A) Pb^2+^ and (B) Cu^2+^ adsorption.

In order to evaluate the practical application value of the Mxene/alginate composites, the regeneration performance of the Mxene/alginate composites was examined in this study (using 0.1 M nitric acid solution as a desorption agent), and the recyclability of the composite adsorbents is depicted in Fig. 12. It is noteworthy to mention that the gelling of sodium alginate is mainly achieved by the exchange of sodium ions from the G residues with the divalent cations. The divalent cations bind to different chains of G blocks to form a structure like an “egg box,” resulting in a three-dimensional network between the cross-linking of different chains. This enables the alginate to have better mechanical properties.^30^ When the Mxene/alginate composite was cross-linked, its regeneration performance was significantly improved compared with that of the uncross-linked Mxene/alginate composite. After four cycles, the adsorption rate was decreased significantly for the uncross-linked Mxene/alginate composite to Pb^2+^ and Cu^2+^. In this study, the adsorption loss rates of the cross-linked Mxene/alginate composite for Pb^2+^ and Cu^2+^ were 8.9% and 5.4% after ten cycles, respectively. These values are lower than those for the uncross-linked Mxene/alginate composite, which were 13.1% for Pb^2+^ and 12.7% for Cu^2+^. This may be because the composite structure has been damaged after repeated adsorption–desorption cycles. After cross-linking with calcium ions, the structural stability of the composites was improved. Thus, the adsorption loss rate had a significant decrease.

## 4. Conclusions

In this study, the Mxene/alginate composites with high adsorption capacity and short equilibrium times were investigated as a high-performance adsorbent for Pb^2+^ and Cu^2+^ removal. Its two-dimensional layered structure and abundant active adsorption sites enable the Mxene/alginate composite to achieve the maximum adsorption capacity for Pb^2+^ and Cu^2+^ at 382.7 and 87.6 mg g^−1^, respectively, and reach the adsorption equilibrium in 15 min. Furthermore, the Mxene/alginate composites can be regenerated through a simple acid treatment and used without apparent loss in performance after cross-linking. The high capacity and efficiency, low temperature sensitivity, and simple regeneration treatment render the Mxene/alginate composites a promising adsorbent for heavy metal ions.

## Conflicts of interest

The authors declare no conflict of interest.

## Supplementary Material
